# Internal migration and the health of the returned population: a nationally representative study of China

**DOI:** 10.1186/s12889-015-2074-x

**Published:** 2015-07-28

**Authors:** Luwen Zhang, Shuaishuai Liu, Guoying Zhang, Shaolong Wu

**Affiliations:** Department of Health Policy and Management, School of Public Health, Sun Yat-sen University, Guangzhou, China; School of Foreign Studies, Guangzhou University of Chinese Medicine, Guangzhou, China; School of Public Administration, South China Normal University, Guangzhou, China; Sun Yat-sen Center for Migrant Health Policy, Sun Yat-sen University, Guangzhou, China

**Keywords:** Migration, Returned population, Physical health, Psychological health

## Abstract

**Background:**

China had 236 million internal migrants in 2012 and the majority of them migrated from rural to urban areas. The research based on medical and epidemical records found that the migrants had worse health than the urban residents, but the household and working place investigations reported better health status. The sick or unhealthy migrants are likely to return to their hometowns, which in turn may cause a report bias or over-estimation of the health status of rural-to-urban migrants in China. This paper explores the association of migration status and the physical and psychological health of Chinese internal migrants.

**Methods:**

Nationally representative household survey data from the China Labor-force Dynamics Survey 2012 (CLDS) were used to analyze the association between the migration status and the health status of internal migrants in China. Migration status of the respondents was measured by hukou status and migration experience and all respondents were divided into four groups: returned population, migrant population, urban residents, and rural residents. Health status of respondents was measured by self-reported physical and psychological health.

**Results:**

Migration experience was associated with the physical health of the returned population. The physical health of the returned population was worse than the migrant population and was distinguished by age and sex. The physical health status of migrant population was significantly better than rural residents, but not significantly better than urban residents. However, the association between migration status and psychological health was not statistically significant. Besides migration status, the socioeconomic status (SES) had a positive correlation with both physical and psychological health status, while occupational hazards exerted negative influence.

**Conclusion:**

The results indicate a tight association between migration experience and health status. The internal unhealthy migrants were more likely to return to their hometown and the migrant population might have limited health advantage.

## Background

Globalization represents not only the global circulation of production factors and commodities, but also the global flow of workforces. According to the *2009 Human Development Report*, there are 940 million immigrants all over the world, and 200 million out of them are international migrants [1]. Literature on immigrants from developing countries to developed countries notes that the immigrants have better health than the natives at their time of first arrival [2, 3]. Selective migration is the major reason for the initial health advantage. Healthy people are more likely to migrate and the host states may prefer healthier people [4, 5]. However, the immigrant health advantage diminishes over time substantially [6]. Eventually, the sick or unhealthy immigrants return to home state, which may possibly cause the statistical bias of immigrants’ health status [7].

The interaction between health and international migration is known as the “Healthy Migrant Effect”. Three hypotheses are used to test nations and subgroups for this phenomenon: (1) The health of immigrants is better than the local residents in the destination place or original place [8]; (2) The health advantage of immigrants dissipates over time [3, 9]; (3) The sick or unhealthy immigrants are likely to return to their hometown [10, 11]. If hypothesis 3 is tenable, it can be inferred that the physical and psychological health status of returned immigrants should be worse than the un-returned immigrants in the host countries.

Hypotheses 1 and 2 have already been proved by a number of studies [12], but few studies have been done to compare the health status (both physical and psychological) of returned migrants and migrant populations in the international investigations to test hypothesis 3. Due to the difficulty in tracing international migrants, this paper mainly aims to test hypothesis 3 by analysing the association between migration status and health status of internal migrants. Hypothesis 1 is also tested and discussed in this research.

China has the world’s largest internal migrant population—236 million in 2012 according to the *2013 Report on China's Migrant Population Development*, and the majority of them migrate from rural to urban areas. The urban residents generally enjoy better health status than the rural residents, but the internal migration in China generated some very interesting issues. The Healthy Migrant Effect has been proved to exist in China’s internal migration and both hypothesis 1 and hypothesis 2 were tested by previous studies [6, 13]. Similar to the international migrant studies, hypothesis 3 has not been proved by internal migrant studies in China.

Actually, the present epidemiological literature and social science literature on the health of internal migrants in China seems to report contradictory findings. The research based on medical and epidemical records claims that migrants have serious health problems—they are vulnerable to communicable and sexually transmitted diseases, occupational injuries and diseases, bad regeneration health, and had higher maternal mortality rate [14]. However, the household and working place investigations in host cities show that the migrants reported better self-evaluated health status than the urban residents [6, 13].

Why did the two data sources generate such completely different findings? One possible explanation is that the sick or unhealthy migrants, who are treated and recorded in medical and public health facilities, are rarely sampled by the household and working place investigations. The sick or unhealthy migrants have returned to their hometown [10], which may cause the reporting bias or over-estimation of the health status of internal migrants in China. Of course, the reasons migrants return to their hometown are diverse and complicated, such as taking care of the family, giving birth, farming or working [15, 16]. Based on the previous study and hypothesis 3, however, we infer that health status is a very important contributing factor to the decision to return in the migrant population [10].

As noted elsewhere, internal migrants in China suffer severe psychological problems due to pressures from 3D (dirty, dangerous and difficulty) work and other factors [17]. Studies on the psychological conditions of internal migrants in China also report inconsistent findings.

Some research has reported worse psychological health status of migrants compared with urban residents [18], but the others argue that the psychological health status of different migrant groups varies depending on the group [19]. But there has been little obvious evidence indicating the psychological health status of migrants that have returned to their hometowns.

As psychological health is closely related to physical health [20], we could assume that the retuned migrants are of worse psychological status than the un-returned migrants. Using data from a nation-wide representative survey in China, this paper aims to test hypothesis 1 and 3 of the Healthy Migrant Effect on both the physical and psychological health of internal migrants.

## Methods

Data from the China Labor-force Dynamics Survey 2012 (CLDS) were drawn to analyze the association between the migration status and the health status of internal migrants in China. CLDS is a nationally representative, multidisciplinary household survey conducted by Sun Yat-sen University, which is an open access database and publicly available at http://css.sysu.edu.cn/.

The research team applied to the CLDS program and received official permission to use the data. CLDS covers a series of topics, such as demography characteristics, family, education, employment, work history, income, migration and health, et al. The subjects of CLDS are the laborers (all family members between the ages of 15-64) from the sample households which are randomly selected from 29 provinces in China (Hong Kong, Macau, Taiwan, Tibet and Hainan are excluded). The national baseline investigation was conducted in 2012 and 3/4 sample communities were investigated^1^. More than 800 investigators collected 303 village questionnaires, 10612 family questionnaires, and 16253 individual questionnaires. All investigators were trained and tested before investigation and were monitored during the investigation. Computer Assisted Personal Interviewing (CAPI) technology was adopted to control data quality.

### Sampling

The survey adopted a multi-stage, multi-stratified, Probability Proportionate to Size Sampling (PPS) sampling method. The first step was to establish the provincial sampling frames. Six provincial sampling frames were established following the geographic definition of east, west and middle region, along with the population size, large and small population of each province. The second step was to sample the counties. In this stratification, all counties were ranked by GDP per capita, and the primary sampling unit was chosen by systematic sampling from random starting point according to the size of the labor force. The third step was to sample the rural and urban communities. All communities administrated by the sampled counties were ranked by the population size, and PPS was performed according to the scale of labor force from each communities. The last step was to extract the households from communities. The mapping-address method was used to establish the last sampling frame. The household address was chosen by the circulated distance sampling method beginning from a random starting. Finally, the laborers of the household (aged from 15 to 64) were included in the individual sample.

### Sample size

To maximize statistical power and meet budget constraint, at least 400 communities were sampled. The best family number in each community was decided by the ratio of community expenditure and household expenditure (C/C_1_) and the variance (σ^2^) of secondary sampling unit (SSU). C = 3000, C_1_ = 700, σ = 0.05, according to Cochran [21] and Raudenbush [22], we determined the best household number as

$$ {\mathrm{n}}_{\mathrm{opt}}\kern0.5em =\kern0.5em 2\sqrt{\frac{\mathrm{c}}{{\mathrm{c}}_1{\upsigma}^2}}\kern0.5em =\kern0.5em 2\sqrt{\frac{3000}{700\times 0.05}}\kern0.5em \approx \kern0.5em 19 $$

Given 10 % tracing loss and the requirement of multi-stage, multi-variable analysis (1.7 times single-stage and single-variable analysis), the final household number within one community was n = 19*1.7/0.9 = 35. So the total household number should be no less than N = 400*35 = 14000. The number of respondents analysed in our research was 15992, with 261 unqualified ones excluded because of missing values.

### Variables and coding

WHO defined health as a state of complete physical, mental and social well-being, and not merely the absence of disease or infirmity [23]. Released by the Canadian government in 1974, and acknowledged worldwide, the Lalonde Report first identified the four “health fields” influencing an individual’s health: health biology (all aspects of human health, physical and mental), environment, life style, and health care organizations [24]. Based on the Lalonde Report’s framework, we chose physical health status and psychological health status as the dependent variables, migration status as the independent variable, and demographic characteristics, working environment, socioeconomic status (SES), health behaviors and healthcare services access as control variables. The control variables were designed to measure the four health fields.

Physical and psychological health status was measured respectively in the CLDS questionnaire. For physical health status, respondents were asked the question “In general, how would you rate your overall physical health?” An answer of “excellent/very good/good” was coded 1 while “fair/poor” was coded 0. For psychological health status, respondents were asked “Did emotional problems (for instance, depression or anxiety) affect your daily work or activities?” The answer “none/few/sometimes” was coded 1, and “frequently/always” was coded 0.

According to migration experience, the respondents were categorized as migrants and non-migrants. Migrants could be further divided into “migrant population” and “returned population” by Chinese household registration system (hukou). In this research, migrant population is defined as the rural population who were now living or working out of their registered hometown for over 6 months when participating in the survey. The “returned population” refers to those who were living in their hometown but had had the experience of working out of the registered county for over 6 months. In terms of the hukou system, the non-migrant population was divided into the “urban residents” and “rural residents” who had never lived or worked out of their registered hometown. In this term, four population groups were identified and analysed in this study.

The demographic characteristics include sex (female = 0, male = 1), age (analysed as continuous variable), marriage (single or divorced = 0, married = 1) and education (primary school or lower = 0, junior high school = 1,senior high school = 2,college or above = 3).

Occupational hazard exposure was used to measure the working environment (not exposed = 0, exposed = 1).

The socioeconomic status of interviewees was self-evaluated using the 10-level Likert Scale. Health behaviors include smoking (do not smoke = 0, smoke = 1) and drinking alcohol (do not drink =0, drink =1).

Healthcare access included financial accessibility (without insurance = 0, with insurance = 1) and geographic accessibility (distance to nearest medical facilities from home, kilometres).

### Models

Multiple logistic regression analysis was performed to explore the association of migration status and physical health and psychological health by controlling the other factors. A total of 5 regression models were established for the total population and subgroups respectively. Model 1 was designed for the total population, model 2 for males, model 3 for females, model 4 for age ≤ 45, and model 5 for age > 45. Considering that physical health and psychological health interact with one another [20, 25, 26], the psychological health status was controlled in physical status analysis as an independent variable, and vice versa.

The software Stata 11 was used to conduct the statistical analysis. The sampling process considered the investigation design effect, therefore the “Svy” order was adopted to set out sampling parameters and to adjust the systematic error caused by sampling design. The weighted means, ratio and standard errors of variables were reported in descriptive results, so did the weighted parameters in multiple regression analysis.

## Results

### Descriptive results

The weighted results are shown in Table 1. 90.91 % of respondents reported “excellent/very good/good” physical health status, and 96.31 % reported “none/few/sometimes” psychological condition. Of the total population, 29.66 % were urban residents, 45.89 % rural residents, 15.18 % returned population, and 9.27 % migrant population. The average age of total respondents was 37.55, with 69.48 % younger than or equal to 45 years old, 50.99 % male, and 75.80 % married. As to education, a majority of the subjects (46.55 %) graduated from junior-high school, 23.63 % from primary school or less, 18.68 % from senior-high school, and only 11.13 % graduated from college or university. The average score for socioeconomic status was 4.08. A total of 57.10 % of respondents had been exposed to occupational hazards. 30.93 % of respondents smoked, 25.57 % drank alcohol, and 87.59 % were covered by medical insurance. The average of distance to the nearest medical facility was 1.23 kilometers.

**Table 1:** Descriptive results of variable, CLDS 2012

Variables (N = 15992)	Percentage/mean	Standard error
Self-evaluated physical health status (%)		
Fair/poor	9.09	0.0063
Excellent/very good/good	90.91	0.0063
Self-evaluated psychological health status (%)		
Frequently/always	3.69	0.0027
None/few/sometimes	96.31	0.0027
Migration status (%)		
Urban residents	29.66	0.0337
Migrant population	9.27	0.0111
Return population	15.18	0.0136
Rural residents	45.89	0.0310
Education (%)		
Lower than Middle school	23.63	0.0150
Middle high school	46.55	0.0122
Senior high school	18.68	0.0108
College or above	11.13	0.0124
Age (mean & %)	37.55	0.2908
>45	30.52	0.0091
≤45	69.48	0.0091
Gender (%)		
Male	50.99	0.0060
Female	49.01	0.0060
Marital status (%)		
Married	75.80	0.0087
Single or divorced	24.20	0.0087
Health insurance (%)		
Not exposed	12.41	0.0085
Exposed	87.59	0.0085
Occupational hazards exposure (%)		
No	42.90	0.0114
Yes	57.10	0.0114
Smoking (%)		
No	30.93	0.0067
Yes	69.07	0.0067
Drinking (%)		
Yes	25.57	0.0076
No	69.07	0.0076
Self-rated SES (1-min, 10-max, mean)	4.08	0.0476
Distance of medical facilities(km, mean)	1.23	0.1095

Survey design effects (strata, cluster, family, and individual weight) were adjusted in the mean and proportion estimations

### Migration status and physical health

The results of the logistic regression of the physical health model were reported in Table 2. The results support the hypothesis that the sick or unhealthy migrant population returned to their hometown (hypothesis 3). In addition, the association between migration status and physical health is distinguished by age and sex. Using the migrant population as reference group, it reveals in the model of total population (OR = 0.661, P < 0.05), model of female (OR = 0.552, P < 0.05), and model of age ≤ 45 (OR = 0.648, P < 0.05) that the physical health of the returned population was significantly worse than the migrant population when controlling the other variables. However, the difference was not shown in the model of the male and model of age > 45.

**Table 2:** Multivariate logistic regression results of self-evaluated physical health, CLDS, 2012

Independent variables	Total population (n = 15992)			Male (n = 7595)			Female (n = 8397)			Age ≤ 45 (n = 8805)			Age > 45 (n = 7187)		
	OR	95 % CI		OR	95 % CI		OR	95 % CI		OR	95 % CI		OR	95 % CI	
Self-evaluated psychological health (Ref: frequently/always)	5.639^***^	4.170	7.627	6.499^***^	4.122	10.248	5.103^***^	3.604	7.226	6.326^***^	4.124	9.703	5.275^***^	3.718	7.482
Migration status (Ref: migrant population)															
Urban residents	1.224	0.887	1.689	1.220	0.761	1.956	1.204	0.763	1.901	0.762	0.472	1.229	1.320	0.845	2.063
Return population	0.661^*^	0.480	0.910	0.772	0.505	1.182	0.552^*^	0.346	0.880	0.648^*^	0.423	0.994	0.814	0.491	1.347
Rural residents	0.756^*^	0.573	0.998	0.858	0.562	1.309	0.681	0.452	1.027	0.740	0.512	1.071	0.733	0.466	1.153
Age	0.937^***^	0.930	0.944	0.937^***^	0.928	0.946	0.935^***^	0.925	0.945	−−	−−	−−	−−	−−	−−
Gender (Ref: female)	1.164	0.890	1.523	−−	−−	−−	−−	−−	−−	1.051	0.680	1.625	1.183	0.906	1.546
Marital status (Ref: single/divorced)	1.337^**^	1.078	1.658	1.434^*^	1.077	1.910	1.208	0.907	1.609	0.723^*^	0.532	0.983	1.374	0.980	1.926
Education (Ref: Primary school & lower)															
Middle high school	1.629^***^	1.402	1.893	1.615^***^	1.269	2.057	1.594^***^	1.300	1.954	2.681^***^	2.040	3.524	1.626^***^	1.346	1.964
Senior high school	2.292^***^	1.749	3.004	2.493^***^	1.742	3.568	2.073^***^	1.435	2.995	4.765^***^	3.062	7.415	2.292^***^	1.700	3.091
Collage and above	2.934^***^	1.948	4.419	3.803^***^	2.065	7.004	2.252^**^	1.347	3.764	5.298^***^	3.192	8.794	3.603^***^	2.128	6.100
Socioeconomic status	1.175^***^	1.123	1.229	1.201^***^	1.138	1.267	1.153^***^	1.088	1.222	1.204^***^	1.135	1.277	1.147^***^	1.091	1.205
Occupational exposure (Ref: not exposed)	0.832^*^	0.707	0.979	0.681^***^	0.553	0.839	0.967	0.784	1.194	1.073	0.848	1.357	0.720^**^	0.575	0.902
Smoking (Ref: do not smoking)	1.074	0.888	1.299	1.075	0.860	1.343	0.942	0.578	1.536	1.067	0.750	1.516	0.993	0.804	1.225
Drinking (Ref: do not drinking)	1.618^**^	1.223	2.141	1.929^***^	1.472	2.527	0.776	0.478	1.260	1.432	0.829	2.472	1.695^**^	1.264	2.271
Health insurance (Ref: without insurance)	0.784^*^	0.616	0.998	0.664^*^	0.460	0.958	0.875	0.642	1.192	0.631^*^	0.436	0.914	0.861	0.645	1.150
Distance to medical facilities	0.951^**^	0.921	0.982	0.960	0.920	1.002	0.942^**^	0.908	0.977	0.962^*^	0.927	0.999	0.944^**^	0.907	0.982

Survey design effects (strata, cluster, and individual weight) were adjusted in the model estimations. B = b coefficient. SE = standard error. ^*^p < 0.05, ^**^p < 0.01, ^***^p < 0.001

The health status of the migrant population was significantly better than the rural residents only in the model of total population (OR = 0.756, P < 0.05). However, the association of physical health status between the migrant population and urban residents was not significant in five models. These findings indicate that, at least in this research, the hypothesis of better health status of the migrant population (hypothesis 1) has only limited support.

We also used the urban residents as reference group and the results showed that the returned population and rural residents all had worse physical health than the urban residents in the model of total population, model of female and model of age > 45. This finding was not presented in the table because of the space limitation.

For the control variables, age had a negative relation to physical health status in the model of total population (OR = 0.937, P < 0.001), of male (OR = 0.937, P < 0.001), and of female (OR = 0.935, P < 0.001). Married migrants enjoyed better health status than the unmarried in the model of total population (OR = 1.337, P < 0.01), and model of male (OR = 1.434, P < 0.05), but negative in model of age ≤ 45 (OR = 0.723, P < 0.05). Respondents having a junior-high school or higher education have much better physical health status than those who attended, or attended but did not finish, primary school. The socioeconomic status had a positive correlation with physical health status in all five models (OR = 1.175, P < 0.001; OR = 1.201, P < 0.001; OR = 1.153, P < 0.001; OR = 1.204, P < 0.001; OR = 1.147, P < 0.001), while occupational hazards exerted negative influence in model of total population (OR = 0.832, P < 0.05), of male (OR = 0.681, P < 0.001), and of age > 45 (OR = 0.720, P < 0.01) . For the health behaviours, smoking was not significantly related to physical health status, but drinking alcohol showed positive influence in the model of total population (OR = 1.618, P < 0.01), of male (OR = 1.929, P < 0.001), of age > 45 (OR = 1.695, P < 0.01). Medical insurance coverage had negative association with physical health status in the model of total population (OR = 0.748, P < 0.05), of male population (OR = 0.664, P < 0.05), and of age ≤ 45 (OR = 0.631, P < 0.05), indicating adverse selection—unhealthy people preferred to buy medical insurance. The distance to medical facilities exerted negative influence, which suggested geographic accessibility might influence health.

### Migration status and psychological health

Using the migrant population as reference group, the psychological health of the returned population is not distinguished from the migrant population in the five models. This finding did not support hypothesis 3. Nor did the psychological health distinguish between the migrant population and rural residents, or between the migrant population and urban residents, which meant hypothesis 1 was not tenable with regard to psychological health. The results indicated that migration status was not associated with psychological health status.

When we used the urban residents as reference group, the results showed no difference of the psychological health among the urban residents, returned population and rural residents in five models (Table 3).

**Table 3:** Multivariate logistic regression results of self-evaluated psychological health, CLDS, 2012

Self-evaluated psychological health	Total population (n = 15992)			Male (n = 7595)			Female (n = 8397)			Age ≤ 45 (n = 8805)			Age > 45 (n = 7187)		
	OR	95 % CI		OR	95 % CI		OR	95 % CI		OR	95 % CI		OR	95 % CI	
Physical health(Ref: fair/poor)	5.758^***^	4.297	7.716	6.734^***^	4.423	10.251	5.146^***^	3.627	7.302	6.444^***^	4.235	9.805	5.281^***^	3.724	7.489
Migration status(Ref: migrant population)															
Urban residents	0.771	0.482	1.234	0.608	0.244	1.512	0.936	0.597	1.469	0.725	0.418	1.259	0.776	0.384	1.569
Returned population	0.712	0.437	1.159	0.540	0.238	1.227	0.953	0.526	1.725	0.816	0.437	1.522	0.564	0.283	1.127
Rural residents	0.867	0.568	1.324	0.818	0.369	1.813	0.906	0.579	1.417	0.947	0.557	1.608	0.754	0.407	1.395
Age	0.992	0.983	1.001	0.994	0.979	1.009	0.990	0.978	1.002						
Gender (Ref: female)	1.391^*^	1.034	1.871							1.458	0.962	2.211	1.302	0.844	2.006
Marital status (Ref: single/divorced)	1.549^**^	1.141	2.102	1.644^*^	1.006	2.688	1.406	0.969	2.041	1.307	0.880	1.941	1.890^**^	1.244	2.873
Education (Ref: Primary school& lower)															
Middle high school	1.446^**^	1.106	1.890	1.520^*^	1.028	2.246	1.365	0.939	1.984	1.487	0.992	2.227	1.379	0.985	1.931
Senior high school	1.605^*^	1.096	2.350	1.436	0.857	2.404	1.765^*^	1.065	2.923	1.585	0.968	2.595	1.814^*^	1.080	3.048
Collage and above	4.083^***^	2.273	7.335	6.342^***^	2.616	15.375	2.854^**^	1.433	5.685	4.751^***^	2.401	9.399	2.365	0.744	7.525
Socioeconomic status	1.103^**^	1.041	1.170	1.112	1.012	1.222	1.093^*^	1.017	1.175	1.056	0.970	1.150	1.156^***^	1.074	1.245
Occupational exposure (Ref: not exposed)	0.693^**^	0.546	0.879	0.583	0.380	0.893	0.798	0.608	1.048	0.584^**^	0.425	0.803	0.802	0.591	1.089
Smoking (Ref: do not smoking)	0.768	0.564	1.046	0.795	0.557	1.135	0.631	0.386	1.031	0.763	0.463	1.260	0.775	0.550	1.091
Drinking (Ref: do not drinking)	1.061	0.774	1.454	0.937	0.675	1.301	1.541	0.754	3.148	1.089	0.678	1.751	1.038	0.741	1.455
Health insurance (Ref: without insurance)	1.140	0.800	1.625	1.546	0.909	2.629	0.868	0.553	1.364	0.968	0.587	1.598	1.462	0.897	2.382
Distance to medical facilities	1.014	0.952	1.079	0.988	0.923	1.058	1.046	0.976	1.120	1.022	0.948	1.101	1.000	0.931	1.074

Survey design effects (strata, cluster, and individual weight) were adjusted in the model estimations. B = b coefficient. SE = standard error. *p < 0.05, **p < 0.01, *** p < 0.001

It also reveals that males had better psychological status than females in the model of total population (OR = 1.391, P < 0.05). Marriage was proved positive in models of total population (OR = 1.549, P < 0.01), of males (OR = 1.644, P < 0.05), and of age > 45 (OR = 1.890, P < 0.01). Education was of significant influence in all 5 models. Socioeconomic status imposed positive influence in the model of total population (OR = 1.103, P < 0.01), of female (OR = 1.093, P < 0.05) and of age > 45 (OR = 1.156, P < 0.001), but exposure to occupational hazards showed negative influence on psychological health in the model of total population (OR = 0.693, P < 0.01) and of age ≤ 45 model (OR = 0.584, P < 0.01). Occupational hazard was negatively associated with both physical and psychological health status, which was supposed to be an influential intervening predictor.

## Discussion

The relationship between migration and health is complicated. Healthy migrant effect is suitable for Chinese internal migration. According to the analysis, the physical health of the returned population was worse than the migrant population. The hypothesis that the sick or unhealthy migrant populations return to their hometown can be supported by their self-evaluated physical health status. The physical health status of migrants is significantly better than rural residents, but not significantly better than urban residents. This finding partly supported hypothesis 1. The analysis of psychological health showed that there did not exist significant association between migration status and psychological health. This research makes a preliminary explanation for the fact that the physical health status of the returned population is worse than the migrant population. The migrant population, however, does not necessarily have better physical health than the urban residents.

The health status of the returned population and migrant population were distinguished in terms of age and sex, which facilitates the understanding of the complex relationship between migration and health [6]. For the female respondents, the returned population had worse physical health status than the migrant population, but male respondents did not report this difference. The first reason that may cause this situation is the roles of men and women in social life in China. Men’s duty is to earn the bread and raise a family, and women’s role is homemaker and takes care of the family [27]. When man’s health is worse, instead of returning to his hometown, a man has to contribute to his family by hanging on to his job in urban areas. However, an unhealthy woman may return to her hometown to contribute to the family by taking care family members and involving herself in agricultural activities. The second reason is the differentials of men’s and women’s social network and social support in urban areas. As comparison with men, most migrant women are in isolated situations with limited opportunities to build social networks [28]. Once the men’s and women’s health become worse, the men might get more social support than women in the urban areas and the women with limited social network in urban areas have to return hometown to seek for social support.

The health status of the returned population aged ≤ 45 is worse than the migration population aged ≤ 45. This may be because the job-related health selection forced laborers aged ≤ 45 to return to their hometowns if their health conditions were not competent for the hard work in urban areas. However, there exists no distinction of health status between the returned population and migrant population aged > 45. It may be due to two reasons: first, the health advantage of migrant population dissipates over time; second, the effect of job-related health selection on those aged > 45 is weaker [29].

The self-evaluated physical health status of the migrant population was not significantly different from urban residents, which suggests that the migrant population may not possess the health advantages as compared to urban residents. This finding is inconsistent with hypothesis 1 and previous studies. It may be due to the weakened health selection caused by short-distance migration. In the previous studies, as the target destinations were metropolitan areas such as Beijing and Hangzhou [6, 13], the migration population had to travel a long distance to migrate; while in the CLDS survey, the subjects were sampled nationwide, and the cross-county short-distance migration weakened the health selection effect. Rather, the finding that health status of migrant population is better than rural residents supports the hypothesis that the migrant population has a health advantage as compared to rural residents from the aspect of labour migration [8, 13].

The association of migration and heath proved by the nation-wide survey data have very strong policy implications. First of all, the outflow of healthy rural residents and the inflow of the unhealthy returned population imposed extra costs on the New Cooperative Medical Scheme (NCMS) [30]. The increased number of the unhealthy population may further burden or even bankrupt the fund. To cope with the risk, it requires the central government to fully consider the comprehensive health condition of the insured, and adjust the capitation subsidy policy by the health requirements of the enrolees by NCMS.

Second, effective policy interventions should be taken to prevent the possible damages to the health of the migrant population. Since exposure to occupational hazards exerts significant influence on the physical and psychological health of the returned population. Very strict regulation should be made to improve the workplace environment and increase occupational protection. Third, since the geographical accessibility is of significant influence on health status, it is therefore necessary to further promote the development of primary health care facilities.

The limitations in this study are as follows. First, the cross-sectional data only reveals the association between migration status and health status, but it does not explain the causality of migration and health. In order to make further exploration, it requires cohort data to analyse the changes among migration status and physical, psychological health, and find the causality influence between migration and health.

Second, only two items of CLDS questionnaire were used to measure the self-evaluated physical and psychological health in this study. Although these measurements have been proved effective in China, further study should employ more objective items and health indicators to measure the health of migrants. Third, this research focuses on the job-related migration and health status of the labour force. Studies on the relationship between un-job-related migration and health are worth doing in the future. Finally, although hypothesis 3 is supported by Chinese internal migrants, but the expansion to the international migration is challengeable. It requires large transnational survey and captures a lot of returned migrants, which is difficult and expensive.

## Conclusion

By using the nation-wide representative survey data from CLDS, this paper aims to test two hypotheses of the Healthy Migrants Effect: the sick or unhealthy immigrants are more likely to return to their hometown, and the health status of migrants is better than the local residents in the destination place or the original place. The results of multiple logistic regression analysis indicated that the physical health status of the returned population was significantly worse than the migrant population controlling other factors.

The association between migration status and health status was distinguished in age and sex. However, the association of psychological health between the returned population and the migrant population was not significant. The findings support the Healthy Migrant Effect hypothesis and had very strong policy implications: the government should improve the capitation subsidy policy of NCMS, exert more strict regulation on the environment of the workplace, and develop primary health care facilities.

## Footnotes

1 To keep representative in each sampling cross section in the rapid urbanization process in China, CLDS has designed a sample rotation solution. The total sample was divided into 4 quarters. In 2012 baseline survey, 3 quarters of total sample was investigated. In 2014, total sample was investigated and 3 quarters of total sample were traced. In 2016, 1 old quarter of sample will be excluded and 1 new quarter of sample will be included. Similarly, 1 quarter of sample will be replaced in each wave in the next survey process.
